# Effect of online formative assessment on summative performance in integrated musculoskeletal system module

**DOI:** 10.1186/s12909-015-0318-1

**Published:** 2015-03-03

**Authors:** Nilesh Kumar Mitra, Ankur Barua

**Affiliations:** 1 Taylor’s University, School of Medicine, No. 1 Jalan Taylors, 47500 Subang Jaya, Selangor Malaysia; 2 International Medical University, Kuala Lumpur, Malaysia; 3 Department of Community Medicine, International Medical University, No.126, Jalan 19/155B, Bukit Jalil, 57000 Kuala Lumpur, Malaysia

**Keywords:** Medical, Students, Web-based, Formative, Summative performance

## Abstract

**Background:**

The impact of web-based formative assessment practices on performance of undergraduate medical students in summative assessments is not widely studied. This study was conducted among third-year undergraduate medical students of a designated university in Malaysia to compare the effect, on performance in summative assessment, of repeated computer-based formative assessment with automated feedback with that of single paper-based formative assessment with face-to face feedback.

**Methods:**

This quasi-randomized trial was conducted among two groups of undergraduate medical students who were selected by stratified random technique from a cohort undertaking the Musculoskeletal module. The control group C (n = 102) was subjected to a paper-based formative MCQ test. The experimental group E (n = 65) was provided three online formative MCQ tests with automated feedback. The summative MCQ test scores for both these groups were collected after the completion of the module.

**Results:**

In this study, no significant difference was observed between the mean summative scores of the two groups. However, Band 1 students from group E with higher entry qualification showed higher mean score in the summative assessment. A trivial, but significant and positive correlation (r^2^ = +0.328) was observed between the online formative test scores and summative assessment scores of group E. The proportionate increase of performance in group E was found to be almost double than group C.

**Conclusion:**

The use of computer based formative test with automated feedback improved the performance of the students with better academic background in the summative assessment. Computer-based formative test can be explored as an optional addition to the curriculum of pre-clinical integrated medical program to improve the performance of the students with higher academic ability.

## Background

Formative assessment consists of activities to determine the level of knowledge of students for the purpose of providing feedback and planning of future instruction [1]. Black & Wiliam [2] suggested that any activity which generates information to be used by the teachers as feedback to modify the teaching-learning activities can be termed as formative assessment [2]. Two core activities of formative assessment described by early researchers [2-4] consist of self-perception by a student regarding the gap between desired knowledge and present state of knowledge and the actions taken by him/her to close the gap. Despite the known benefits of formative assessments, many institutions had been reluctant to integrate such assessments into the medical curriculum [5]. A study conducted among 909 final-year students observed that the students receiving a remedial intervention after poor performance on a formative test were able to improve marks in the summative examination as compared to those who did not receive remediation [6].

Multiple Choice Question (MCQ) format can be easily adopted in the hypermedia environment because of ease of producing fully integrated, automated tests which instantly supply feedback to the participant with suggested direction for further study [7]. Add-on packages can be used to give feedback to the students on incorrect answers giving the reasons for an option to be incorrect. To be effective, such system should be user-friendly and should not place too much cognitive demand on the user. The utility of a formative test is partially dependent on the manner through which the feedback is provided to the learner [8]. Paper-based formative assessment followed by face-to-face feedback in the class often makes the low-achiever feeling bad. A computer-based online formative assessment can be self-administered and it is not possible for the others to know about the performance of an individual [9]. Computer-based assessment with a feedback process is beneficial for the learning process as it provides immediate feedback [10]. Thereby the students take control of their own learning and this stimulates the learning process. Online formative tests can serve as effective test preparation strategy. The positive impact of multiple online formative tests on the outcomes of the summative test has not been clearly established. Cassady & Gridley (2005) in a study conducted in consecutive two cohorts of university level psychology students found no consistent pattern of impact of online formative test on the outcomes of the summative test when using past performance as a covariate [11]. In spite of great enthusiasm among the educators, there is little evidence regarding the impact of web-based assessment practices on student performance [12].

This study was conducted to compare, the effect on performance in summative assessment, of repeated computer-based formative assessment with automated feedback with that of single paper-based formative assessment with face-to face feedback.

## Methods

The designated university in Malaysia offered a five-year medical program (MBBS) in which the pre-clinical curriculum was composed of five semesters (two years and six months) accommodating multiple body-system modules. Each system module ran for five to six weeks. The contents of the module were integrated across the subject disciplines (Anatomy, Physiology, Biochemistry, Pathology, Microbiology, Pharmacology, Clinical skills and Clinical sciences) relevant to the body system. The Musculoskeletal module was offered in semester 5 which preceded the Nervous system module. The summative assessment was held at the end of Nervous system with components from both the Musculoskeletal and Nervous system.

The banding pattern of the students entering in the medical program was computed by the selection committee of the designated university based on the transcript of the pre-university examination. An academic banding stratification is practiced in the university to equate one entry qualification against another. There are several pre-university programs in Malaysia. The designated university also admits international students. Banding rating classifying the students into Band 1, 2, 3 and 4 was developed based on equivalence of grades of the students secured in four science subjects among different pre-university programs.

Different schools under the university started using the web-based learning portal from 2007. The study-guides and the learning resources were placed in the portal according to the schools. Faculty members were facilitated by the staff of the e-learning department to develop online tools to support e-learning and e-assessment. The online formative MCQ tests were placed in the portal according to the modules. All formative quizzes were administered through MOODLE, which the students accessed online by using their personal username and password.

This study was a quasi-randomized controlled trial which used the post-test only comparative study design. Blinding and allocation concealments were not conducted due to feasibility constraints. The sample consisted of students who were enrolled in the Musculoskeletal system module at the designated university. A total of 170 students were enrolled in the Musculoskeletal system. The module was of 5-weeks duration. The students were selected by using the stratified random sampling technique. The distribution of 4 bands of students among the students in group C and group E was almost similar (Table 1) and this was used as a criterion for stratification.

**Table 1:** **Distribution of pattern of banding between the experimental and control groups**

Banding Pattern	Group E	Group C
	(%)	(%)
Band 1	18.5%	19.6%
Band 2	20%	24.5%
Band 3	12.3%	15.7%
Band 4	20%	16.7%

Group C was subjected to a single paper-based formative test consisting of MCQs (one best answer) on the 4^th^ week of the Musculoskeletal system. Group E was exposed to three computer-based formative tests consisting of MCQs (one best answer) on the Musculoskeletal system. Content mapping and item analysis were done to ensure content validity and reliability of the test conditions. Among the selected students of group E, 65 students participated in the online formative test. Among the selected students of group C, 102 students participated in the paper-based formative test. The gender and university entry banding pattern were recorded for each participant.

### Ethical issues

The ethical approval for the study was granted by the Joint Committee of Research and Ethics of the International Medical University (IMU), Malaysia. Informed written consent was obtained from each participant prior to the data collection. Confidentiality of data was maintained throughout this study.

### Summative assessment

In the Musculoskeletal module, students were assessed in a summative examination at the end of semester 5 after 11 weeks (5 weeks of Musculoskeletal and 6 weeks of Nervous system). All the students of the class went through the common end-of-module assessment, which contained MCQs, modified essay questions and objective structured practical examination questions on Musculoskeletal system and Nervous system. There were forty MCQs in the end-of-module assessment. The scores of the students belonging to group C and group E in the MCQ component of Musculoskeletal module in this examination were recorded as the summative examination score to be used in this study. To ensure validity in this summative assessment, the items were selected based on a blue print or table of specification. Item analysis was also done to ensure reliability of the test condition. Since the test questions were thoroughly vetted by the members of module working committee, it was assumed that the test was a valid tool to gauge students’ knowledge in the Musculoskeletal system.

### Face-to-face formative assessments

Single paper-based formative MCQ test was offered on the 4^th^ week for the students of group C. The test consisted of 30 MCQ questions which were designed matching the style and difficulty level of summative examination. The test was time-tabled and administered under supervision. The testing time provided was two minutes per MCQ item. The feedback prepared by the content-lecturers, was provided face-to-face to the group C students immediately after the test by the module coordinator. The performance was reported as percentage. The mean and SD (standard deviation) were computed.

### Online formative assessments

Three online formative MCQ tests were offered during the learning period of Musculoskeletal module on the 2^nd^ week, 3^rd^ week and 5^th^ week for the students of group E. Each online test consisted of 20 MCQs designed to match the style and difficulty of summative examination. The testing time provided was two minutes per MCQ item. Students were made aware of the quizzes in the course syllabus by class announcements. Students’ performance on each test were reported as percentage and the mean marks of 3 tests with SD were computed. Feedback was given automatically by the computer program after every attempt of answering the set of questions. The software used in the online formative test was “Moodle” interactive software.

### Feedback process

Being an integrated system-based module, the MCQ items were provided by the different content-lecturers (Anatomy, Physiology, Pathology, Pharmacology, Clinical Sciences etc.). The items were vetted by the module coordination group. The feedback to the individual incorrect or correct options of MCQ items were constructed by the content-lecturers. The feedback was entered into the quiz section of the ‘Moodle’ software for online formative assessment in the e-learning portal by the module coordinator. After answering the items and selecting the correct options, the students would submit the answers. This would automatically open the feedback against the selected option. The similar items were also used for the paper-based formative test. The feedback was projected in a power-point presentation after the test and discussed by the module coordinator to the students taking the paper-based test. The students were allowed to raise their questions during this feedback session.

### Measures for internal validity

The appearance of the students in the formative tests in both group C and group E students was on voluntary basis. The marks were not computed by the academic section of the university for the transcript. During the previous years, this module had formative tests. Either online or paper based test was used in the previous years. As it was usual practice, students’ lack of awareness about the mode of formative tests did not affect the results of the experiment. The testing environment was not similar in the two groups of students. The students in the online formative test group were taking the computer-based test on their own initiative either in the computer lab or in their own personal computer. The students in the paper-based formative test group were taking the formative test at a pre-selected time and date given in the time-table in an environment controlled by the lecturer.

### Data analysis

The data collected were tabulated and analyzed by using the Statistical Package for Social Sciences (SPSS) version 17.0 for windows. The comparison between the mean scores of summative assessment of group C and group E was conducted by the statistical test of Independent Samples t-test. The relationship between the scores of group C in the paper-based formative test and the summative assessment was done by statistical test of Pearson’s Correlation Coefficient. Similarly the relationship between the scores of group E in the computer-based formative test and the summative assessment was also tested by the Pearson’s Correlation Coefficient. In this study, p-value <0.05 was considered to be statistically significant.

## Results

The overall mean score (male and female students together) in the end-of-module summative assessment was almost similar in the student group exposed to the online computer based formative test and the student group exposed to the paper-based formative test (Table 2). However, among male students, mean score was higher in group E exposed to the online formative test. Among female students, mean score in the summative assessment was higher in group C, exposed to the paper-based formative test. Band 1 students belonging to group E (online formative test group) showed higher mean score in the summative assessment compared to similar band of students in group C (paper based formative test group). Band 4 students belonging to group E(online formative test group) showed higher mean score in the formative test (Table 3) compared to similar band of students in group C. Mean score for this band of students in the summative assessment was lowest.

**Table 2:** **Comparison of summative assessment scores between the experimental and control groups according to gender and banding**

Categories	Subcategories	Group E	Group C
		(Mean ± SD)	(Mean ± SD)
**Gender**	Both Genders	56.4 ± 12.2	56.9 ± 13.6
	Male	60 ± 13.6	56.5 ± 13.5
	Female	53 ± 9.8	57.2 ± 13.7
**Banding**	Band 1	62.5 ± 13.9	58 ± 12.7
	Band 4	51.1 ± 8.7	54.4 ± 13.8

**Table 3:** **Comparison of formative assessment scores between the experimental and control groups according to gender and banding**

Categories	Subcategories	Group E	Group C
		(Mean ± SD)	(Mean ± SD)
**Gender**	Both Genders	61.7 ± 17.6	49.2 ± 12.8
	Male	60.9 ± 21.2	52.6 ± 12.1
	Female	62.5 ± 13.7	46.8 ± 12.8
**Banding**	Band 1	71.8 ± 16.2	52 ± 10
	Band 4	58.2 ± 16.9	46 ± 11.9

The mean score of the online computer based formative test in the Group E was much higher than the mean score of the paper based formative test in Group C. However the variation among the individual scores in Group E was higher, as evident by the higher standard deviation (Table 3). It was found that both male and female students of Group E who went for the online computer based formative test performed better compared to their counterparts in Group C who went for the paper-based formative test. Compared to the difference between Band 1 and Band 4 students observed in the summative scores, both Band 1 and Band 4students had higher mean scores in the computer based formative test.

**Figure 1:**
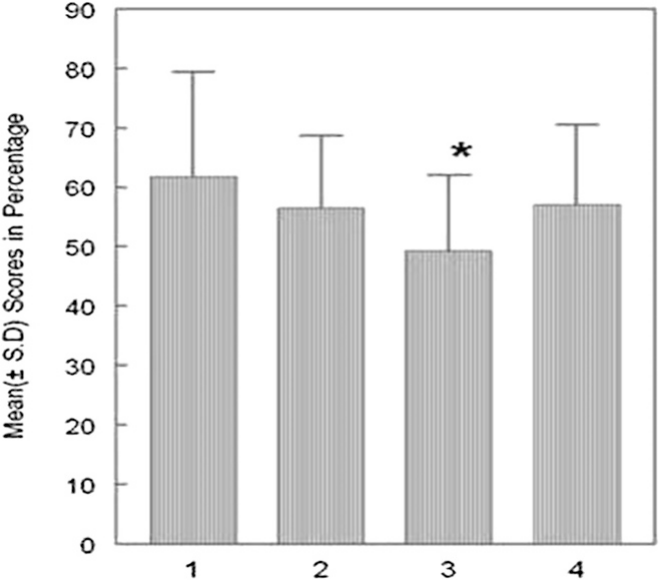
**Bar Chart Comparing the Scores of Both Formative and Summative Assessments.** Here, 1 = Formative score group E; 2 = Summative score group E; 3 = Formative score group C; 4 = Summative score group C; Independent Samples t-test comparison between mean summative scores between Group E and Group C, t(165) = −0.254, p > 0.05; *Independent Samples t-test comparison between mean formative scores between Group E and Group C, t(165) = 5.334, p < 0.05.

The mean score of the formative test in group E exposed to computer-based formative test was significantly higher than that in group C exposed to paper based test [t(165 = 5.334, p < 0.05] (Figure 1). However, the means score of the summative assessment in group E exposed to computer based formative test with online feedback was not significantly different from that in group C exposed to paper-based formative test with face-to face feedback [t(165) =0.254, p > 0.05].

The scores of end-of-module summative assessment in MCQ test of Musculoskeletal system and the formative test scores of the group E exposed to online computer based formative assessment were tested for correlation. The Pearson’s Correlation Coefficient for group E showed a positive (r^2^ = +0.328) and significant (p = 0.008) correlation between the online formative test score and the end-of-module summative test scores. Hence, any increase in the online formative test score would contribute to a small increase (32.8%) in the performance on the summative assessment in MCQ test of musculoskeletal system.

The scores of end-of-module summative assessment and the formative scores of the group C exposed to paper-based formative test were also tested for correlation. The Pearson’s Correlation Coefficient for group C showed a positive (r^2^ = +0.176) and significant (p = 0.076) correlation between the paper-based formative test score and the end-of-module summative test scores. Hence, any increase in the paper-based formative test score would contribute to a small increase (17.6%) in the performance on the summative assessment in MCQ test of musculoskeletal system. The proportionate increase of performance in group E was found to be almost double than group C.

## Discussion

Positive correlation between the scores of computer-based formative test and the scores of summative assessment in the group of students exposed to repeated computer-based formative test indicated that the formative test affected the performance of the students in the summative assessment. Statistical analysis failed to find out significant difference between the mean scores of summative assessment of group C, exposed to the paper-based formative test and group E, exposed to the computer-based formative test. Previous study on biological science undergraduate students found variable impact of formative quizzes on the summative performance. The study compared the summative examination performance before and after introduction of the quizzes. Level 1 Human Physiology and year 2 Neurobiology modules showed improvement in the percent of students getting good marks and decrease in the percent of students failing in the examination. However, in year 3, Parasitology module showed variable percentage of students obtaining good results. The positive influence of computer-based formative quizzes on the summative examination performance was not uniform in all modules. A significant number of students did not use the web-based formative test as it was not compulsory [9].

The students of Band 1 academic pattern who were admitted in the medical program with higher academic grades and were exposed to repeated online formative tests with automated feedback showed comparatively higher mean score in the summative test. Henley & Reid (2001) found in their study that brighter highly motivated students accessed online test materials more often than the weaker students and got benefitted by the test [13]. The more able students could self-regulate their learning process with the help of the online formative test. This was facilitated by more number of questions in the online formative test and more immediate feedback compared to the paper-based formative test. Male students performed better in the online test group while the female students did better in the paper-based test group. This finding might have been produced due to grouping character as female students (57.8%) were more in the paper-based test group compared to the online test group (52.3%). However inferential statistics proved that there was no significant difference between the performances on the summative assessment of the two treatment groups.

The repeated online test helped the students of group E to become test-wise. The mean score of formative test was significantly higher in Group E compared to the mean score of formative test in group C. The online test permitted the students to get automated feedback after submission of chosen option of few items. This made them test-wise resulting in the increase in the mean score of the formative test. Compared to the traditional paper-based assessment, online assessment requires a more systematic and innovative approach to match the level of desired competencies of the students [14]. A positive and significant correlation was found between the score of formative test and the score of end-of-module summative test in the group E exposed to online computer based formative test with automated feedback. The feedback in formative assessment drives the learning process. In this study, group C students received single feedback at 4^th^ week of the module which was face-to-face, time-tabled and supervised by a facilitator. The group E students received online feedback which was multiple, after each of the three formative tests held at 2^nd^ week, 3^rd^ week and 4th week of the module. The students taking the online formative test were at liberty to take the test and the feedback at their own time. The paper-based formative test with face-to-face feedback did not show any significant correlation between the score of formative test and score of summative test. Smith 2007, in a retrospective study with Geosciences undergraduate course found that the online formative quiz scores and summative examination scores correlated strongly (r^2^ = 0.86) [15]. The stronger correlation compared to our study (r^2^ = 0.107) was possibly due to additional in-class assignments and written assignments given to the students in addition to online quiz in the study by Smith.

Two previous studies had analyzed the performance in the final examination scores after giving the online formative quiz. Both these studies were without any control group having an alternative mode of formative test [16,17]. Angus & Watson used a retrospective regression method in a first-year mathematics course in an Australian school of business, enrolled in 2006 (n =397) and 2007 (n =1239) [16]. It was concluded that considering the performance in end-of semester examination, exposure to regular (low-mark) online quiz produced a significant and positive effect on student learning.

In the present study, the students participated voluntarily in the formative tests having no contribution to the final transcript. Conversely the study by Angus & Watson used online quiz which had 2% weight out of overall marks in final examination [16]. One of the possible reasons for lack of motivation of the students to accept the feedback and improve the performance was the absence of weight to the scores of the formative test. The relationship between the student motivation and incentives for formative test was described by the study done by Kibble (2007) [17]. Kibble studied a large (n = 350) Medical Physiology course in a Caribbean medical School with varying incentives (0%, 1%, 1.5%, 2% per quiz). It was found in that study that when the incentives were increased, student participation rose dramatically.

The demographic distribution, which might have affected the motivation of learning through computer based formative test, in this study, was comparatively more percentage of Band 4 students and less percentage of Band 2 students in group E who received online computer based formative test The learning generated by automated feedback could not produce significant difference in the performance of summative assessment of the Musculoskeletal module. The reasons can be traced back to the theory of feedback associated learning by Black & Wiliam [2]. Black & Wiliam commented that even if the feedback comments were operationally helpful for the student, the effect would be undermined by negative motivation. The Musculoskeletal module is an integrated multi-disciplinary module, where difficulty level varies between the subject disciplines (Anatomy, Physiology, Pathology, Pharmacology and Clinical Sciences). The lack of incentives and absence of lecturer-controlled-environment are the factors which might have produced negative motivation in the learning process from software generated feedback system in the online formative test.

### Limitations of the study

Due to reasons related to the academic administration, the number and timing of formative assessment in the control and intervention groups could not be kept as similar in this study. Offering limited incentives like minor grade points to the formative assessment in the study would most likely improve the motivation of the students and affect the performance in the summative assessment positively. Gap of six weeks (of nervous system module) between formative assessment intervention and summative assessment performance was an important factor reducing the direct effect of intervention.

## Conclusion

Conventionally, the performance in summative assessment is accepted as an indicator of learning process. This study revealed that a trivial, but positive association existed between the online computer based formative test with automated feedback and the performance in the summative assessment in a multi-disciplinary integrated module of third year MBBS program. It can be extrapolated that any increase in the use of computer based formative test with automated feedback would produce a small increase in the score of the summative assessment of the student due to improvement in the learning process. The students entering the medical program with higher academic ability would benefit more by the exposure to online computer based formative test. Use of computer-based formative test with automated feedback can be explored as an optional addition to the curriculum of pre-clinical integrated medical program to improve performance of the students with higher academic ability.
